# Bioluminescent Reporting of In Vivo IFN-γ Immune Responses during Infection and Autoimmunity

**DOI:** 10.4049/jimmunol.1801453

**Published:** 2019-02-27

**Authors:** Catherine J. Reynolds, Deborah L. W. Chong, Yihan Li, S. Lucas Black, Amy Cutler, Zoe Webster, Jiten Manji, Daniel M. Altmann, Rosemary J. Boyton

**Affiliations:** * Lung Immunology Group, Infectious Diseases and Immunity, Department of Medicine, Imperial College London, London W12 ONN, United Kingdom; and; † Transgenics and Embryonic Stem Cell Facility, Medical Research Council London Institute of Medical Sciences, London W12 ONN, United Kingdom

## Abstract

IFN-γ is a key cytokine of innate and adaptive immunity. It is important to understand temporal changes in IFN-γ production and how these changes relate to the role of IFN-γ in diverse models of infectious and autoimmune disease, making the ability to monitor and track IFN-γ production in vivo of a substantial benefit. IFN-γ ELISPOTs have been a central methodology to measure T cell immunity for many years. In this study, we add the capacity to analyze IFN-γ responses with high sensitivity and specificity, longitudinally, in vitro and in vivo. This allows the refinement of experimental protocols because immunity can be tracked in real-time through a longitudinal approach. We have generated a novel murine IFN-γ reporter transgenic model that allows IFN-γ production to be visualized and quantified in vitro and in vivo as bioluminescence using an imaging system. At baseline, in the absence of an inflammatory stimulus, IFN-γ signal from lymphoid tissue is detectable in vivo. Reporter transgenics are used in this study to track the IFN-γ response to *Pseudomonas aeruginosa* infection in the lung over time in vivo. The longitudinal development of the adaptive T cell immunity following immunization with Ag is identified from day 7 in vivo. Finally, we show that we are able to use this reporter transgenic to follow the onset of autoimmune T cell activation after regulatory T cell depletion in an established model of systemic autoimmunity. This IFN-γ reporter transgenic, termed “Gammaglow,” offers a valuable new modality for tracking IFN-γ immunity, noninvasively and longitudinally over time.

## Introduction

There has been a strong impetus to generate transgenic mouse strains able to facilitate imaging of adaptive immune responses. This has led to the use of new, transgenic, mouse reporter strains for several cytokines as well as for NF-κB as a marker of transcriptional activation of innate and adaptive immunity. With the exception of bioluminescent reporter NF-κB reporter mice for biophotonic imaging, the majority of strains use fluorescent reporters for two-photon imaging modalities. We set out in this study to generate a reporter strain for in vivo screening of the immune responses involving IFN-γ as an effector cytokine.

IFN-γ is produced by activated lymphocytes, including NK cells, NKT cells, CD4^+^, and CD8^+^ T cells (1), although IFN-γ production by other leukocytes, such as monocytes/macrophages (2), dendritic cells (3) and neutrophils (4), has been described. Increased susceptibility to infection as a consequence of defective expression of IFN-γ or its receptor in both mice (5) and humans (6, 7) highlights a central role for IFN-γ in both viral and bacterial pathogen clearance. Conversely, overexpression of this cytokine has been associated with aberrant inflammation and autoimmunity (8, 9). However, there are many examples of anti-inflammatory actions ascribed to IFN-γ (10), so that the resulting picture is a nuanced one in which the role of IFN-γ is highly context and timing dependent (11).

The ability to monitor IFN-γ production, noninvasively, in an in vivo setting, over extended periods of time would be of enormous value in the study of diverse disease models of infection, tumor immunity, and autoimmunity. Such a modality offers the potential for real-time, noninvasive monitoring of Th1 adaptive immunity.

Many cytokine reporter mice have been generated, the majority of which function by expressing a fluorescent marker under the control of the cytokine gene promoter (12). YETI and GREAT mice are examples of IFN-γ reporters wherein IFN-γ production can be imaged through yellow fluorescent protein expression (13, 14). In both of these lines, the fluorescent marker is targeted to the endogenous IFN-γ locus as a knock-in. An alternative approach employed in some transgenic reporter lines, including an IFN-γ reporter in which IFN-γ^+^ cells are tagged as Thy1.1^+^ (15), is to use a bacterial artificial chromosome (BAC) transgene. A BAC transgenic approach means that it is possible to use extensive, endogenous promoter and enhancer elements to faithfully report expression patterns from the gene locus of interest.

Cytokine reporter mice generated to date are not suited to in vivo bioluminescence reporting of IFN-γ immunity. Common approaches for in vivo imaging studies use bioluminescent molecules and their substrates, such as firefly, *Renilla*, and bacterial luciferases (16). Bioluminescent reporter systems have previously been developed for some other cytokines, such as IL-7 (17) and IFN-β (18). Furthermore, NF-κB/luciferase reporter mice have been invaluable for generic imaging of inflammatory responses and their regulation. Although these mice are valuable for tracking innate immunity or inflammation, none allows the capacity to track type 1 immunity in vivo; a vast array of immunology applications depends on the ability to track IFN-γ responses, be these to virus, bacteria, tumor, fungi, or autoimmunity. Our specific intention was to make this achievable allowing the noninvasive, longitudinal analysis of IFN-γ responses from the earliest stage.

In this study, we report the generation of an IFN-γ reporter mouse in which IFN-γ production can be visualized in vivo as a bioluminescent signal from firefly luciferase. We show that IFN-γ is continuously produced by lymphoid tissue at steady-state and that this model can be used to study IFN-γ production during longitudinal infection studies, to monitor primary immune responses to Ag, and to track the onset of inflammation and tissue infiltration during systemic autoimmunity.

## Materials and Methods

### Modification of IFN-γ BAC clone

**FIGURE 1.**
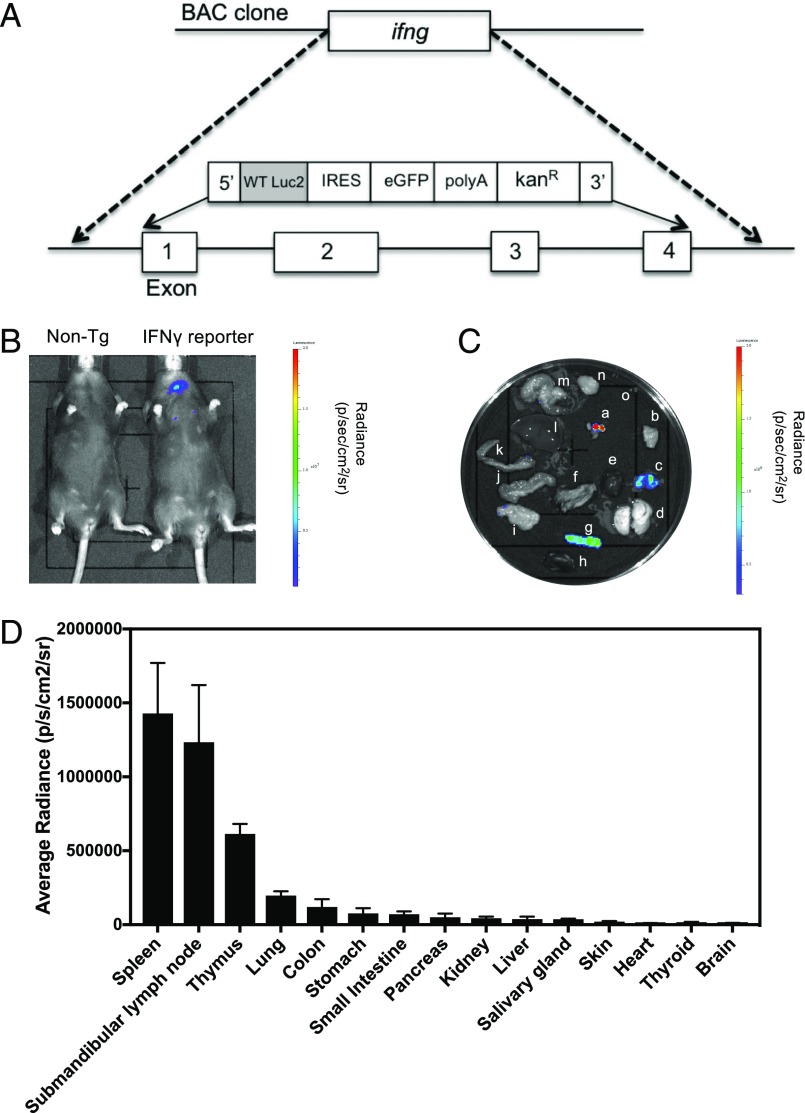
IFN-γ reporter transgenic generated using a modified BAC clone. (**A**) The BAC clone RP24-368M14, containing the coding and promoter elements of the *ifng* gene, was modified such that exons 1–4 of the *ifng* gene were replaced with a reporter construct encoding the firefly luciferase gene (*luc2*), GFP, and a PolyA. (**B**) IFN-γ reporter (*n* = 5) and nontransgenic mice were injected i.p. with 150 mg/kg d-Luciferin XenoLight d-luciferin – K^+^ Salt (PerkinElmer). Ten minutes postinjection, the bioluminescence signal in each mouse was detected using the IVIS imaging system. (**C**) Submanibular lymph nodes (**a**), salivary gland (**b**), thymus (**c**), lung (**d**), heart (**e**), skin (**f**), spleen (**g**), kidney (**h**), pancreas (**i**), small intestine (**j**), colon (**k**), liver (**l**), stomach (**m**), brain (**n**), and thyroid (**o**) were removed and IVIS imaged. (**D**) The bioluminescence signal was quantified by calculating the average radiance of each organ. Error bars represent mean ± SEM. This experiment was repeated on two separate occasions.

BAC clone RP24-368M14 (bacpacresources.org) was modified to replace exons 1–4 of the *ifng* gene with a reporter construct containing coding sequences for the firefly luciferase gene, *luc2* (from imaging vector pGL2; Promega), GFP, a bovine growth hormone polyadenylation signal (PolyA), and a kanamycin resistance gene (Kan^R^) (Fig. 1A). Correct targeting to the *ifng* gene was achieved using a 93-bp 5′ homology arm and a 163-bp 3′ homology arm immediately upstream of *ifng* exon 1 and downstream of *ifng* exon 4, respectively. The BAC clone was modified using the Red/ET recombination method and linearized using PI-SceI prior to pronuclear injection into C57BL/6 × CBA oocytes.

### Genotyping of IFN-γ reporter transgenics

Mice were genotyped by PCR using genomic DNA isolated from ear biopsy specimens. IFN-γ reporter mice were identified by the presence of a 300-bp PCR product using primers specific for the *luc2* gene (forward primer: 5′-ACAAGTACGACCTGAGCAAC-3′; reverse primer: 5′-CTGGTAGCCCTTGTACTTGAT-3′).

### Mice

Transgenic mouse generation, breeding, and experiments were performed within U.K. Home Office legislation under the terms of a project license (Public Project License No. 70/7708) granted for this work under the “Animals (Scientific Procedures) Act 1986.” The founder IFN-γ reporter mouse was crossed onto the C57BL/6 strain to establish the transgenic line. Reporter mice were crossed with Foxp3-DTR–inducible knockout mice (19) to allow regulatory T cell (Treg) depletion experiments. The mice were then bred to homozygosity for the reporter construct.

### In vivo and in vitro bioluminescence imaging

Mice were injected i.p. with 150 mg/kg XenoLight d-luciferin – K^+^ Salt (PerkinElmer, U.K.). Mice were left for 10 min prior to the induction of anesthesia using isoflurane and bioluminescence measurement using an IVIS Lumina II Imaging System (PerkinElmer). Average radiance for areas of interest in bioluminescence images were calculated using Living Image 4.2 Software (PerkinElmer). For an in vitro measurement of bioluminescence using cultured cells, cells were transferred to black 96-well tissue culture plates before the addition of d-luciferin at a final concentration of 150 μg/ml. Cells were incubated for 10 min before imaging.

### In vitro cell culture

Single-cell suspensions of splenocytes from IFN-γ reporter or nontransgenic mice were cultured in RPMI medium containing 10% FCS and 50 μM 2-ME and supplemented with l-glutamine and penicillin–streptomycin. For stimulations with PMA and ionomycin, cells were cultured for 24 h with a 2-fold dilution series starting at 50 ng/ml PMA and 1 μg/ml ionomycin. For polarization experiments, cells were stimulated with anti-CD3 (1 μg/ml) and anti-CD28 (0.5 μg/ml) (eBioscience), with the addition of rIL-2 (10 IU/ml) and rIL-12 (10 ng/ml) for Th1-polarizing conditions and rIL-2 (10 IU/ml), rIL-4 (10 ng/ml), and anti–IFN-γ (10 μg/ml) for Th2-polarizing conditions. All recombinant cytokines were purchased from R&D Systems. Anti–IFN-γ was purchased from Life Technologies.

### Quantification of IFN-γ by ELISA and real-time PCR

IFN-γ in cell culture supernatants or in homogenized lung tissue was quantified by ELISA, using paired Abs specific for mIFN-γ (Mabtech). Lung tissue samples were prepared by homogenizing the lung at a concentration of 100 mg/ml in Hank’s balanced salt solution, without MgCl_2_ or CaCl_2_, containing a protease inhibitor mixture (Roche). For quantification of *ifng* transcripts by real-time PCR, RNA was isolated from the lung tissue using TRIzol (Life Technologies) and reverse transcribed using SuperScript III (Life Technologies). Real-time PCR reactions were performed using Brilliant II QPCR Low ROX Master Mix (Applied Biosystems) with *gapdh* and *ifng* gene-specific primers and probes (Assays-on-Demand Gene Expression assays; Applied Biosystems).

### *Pseudomonas aeruginosa* lung infection

*P. aeruginosa*, strain PA01, was grown in Luria–Bertani broth overnight at 37°C. Mice were anesthetized with isoflurane and infected intranasally with 5 × 10^6^ CFU in sterile PBS. At the point of cull, lung tissue from each mouse was harvested for CFU determination by the plating of homogenized lung samples onto Luria–Bertani agar and colony counting.

### Immunization with *Pseudomonas* OprF Ag

Mice were immunized s.c. in one hind footpad with 1, 5, or 25 μg of rOprF protein (20) emulsified with Hunter “TiterMax Gold” adjuvant (Sigma-Aldrich) or with adjuvant emulsified with PBS alone. At 11 d postimmunization, the draining popliteal lymph nodes were harvested and disaggregated into a single-cell suspension. CD4^+^ T cells responding to OprF Ag were quantified by IFN-γ ELISPOT (Diaclone; 2BScientific) performed in HL-1 serum-free medium (BioWhittaker) supplemented with l-glutamine and penicillin–streptomycin. Cells (2 × 10^5^) plus Ag were added to wells of precoated anti–IFN-γ ELISPOT plates and incubated for 72 h at 37°C with 5% CO_2_. Plates were developed according to the manufacturer’s instructions, and spots were counted on an automated ELISPOT reader (Autoimmun Diagnostika). Replica stimulations were set up in normal tissue culture plates for the measurement of IFN-γ in culture supernatants by ELISA.

### Treg depletion

Tregs in IFN-γ reporter mice that had been crossed onto the Foxp3-DTR transgenic line were depleted by either daily or alternate daily administration (i.p.) of diphtheria toxin (DT) (1.25 μg/kg) (Sigma-Aldrich).

## Results

### Generation of IFN-γ reporter transgenics

We set out to generate a new transgenic mouse model in which the expression of IFN-γ was reportable as a bioluminescent signal. A BAC clone containing regulatory and coding elements of the mouse *ifng* gene was modified such that all four exons of *ifng* were replaced with the coding sequence of the firefly luciferase gene (l*uc2*), GFP, and a PolyA (Fig. 1A). *Ifng* is located on mouse chromosome 10 and is the only gene in the surrounding region; other nearby coding regions are located 145 Kb upstream (Iltifb) and 412 Kb downstream (Dyrk2) of the *ifng* sequence; however, the selected BAC clone (RP24-368M14) is 174 kb in size and does not contain either of these genes. As the entire coding region of *ifng* had been replaced and no other coding regions are present, we anticipated no extraneous biological consequence of expressing the BAC construct, other than the normal positional risk of transgene integration. The modified BAC clone was linearized and injected into C57BL/6 × CBA oocytes to generate an IFN-γ reporter founder mouse. The founder IFN-γ reporter mouse was identified by PCR and crossed onto the C57BL/6 strain to establish the transgenic line.

### Detection of bioluminescent signal at baseline in reporter transgenics

To determine whether a bioluminescence signal was detectable in vivo in healthy mice, IFN-γ reporter mice and control nontransgenic mice were injected with d-luciferin substrate, anesthetized, and imaged using the IVIS Lumina II Imaging System. Reporter mice showed a bioluminescent signal reproducibly detectable in the neck region (Fig. 1B). To determine which internal organs the signal derived from, organs were dissected and imaged ex vivo (Fig. 1C). Quantification of bioluminescence from each organ showed that the signal predominantly derives from lymphoid tissue (spleen, lymph node, and thymus), although a low level of bioluminescence was also detectable in other tissues, including the lung and the digestive tract (Fig. 1D). We therefore conclude that the neck signal present in in vivo images of healthy adult mice derived from lymph nodes in this region and that the signal from the thoracic cavity likely derived from the thymus. The signal localized to spleen was detected ex vivo but not in vivo, suggesting that a beneficial additional gain in sensitivity may be achieved by crossing to an albino strain. Splenocytes were analyzed by flow cytometry for expression of GFP, but this was not detectable.

### Bioluminescence signal in IFN-γ reporter transgenics is linked to IFN-γ production

**FIGURE 2.**
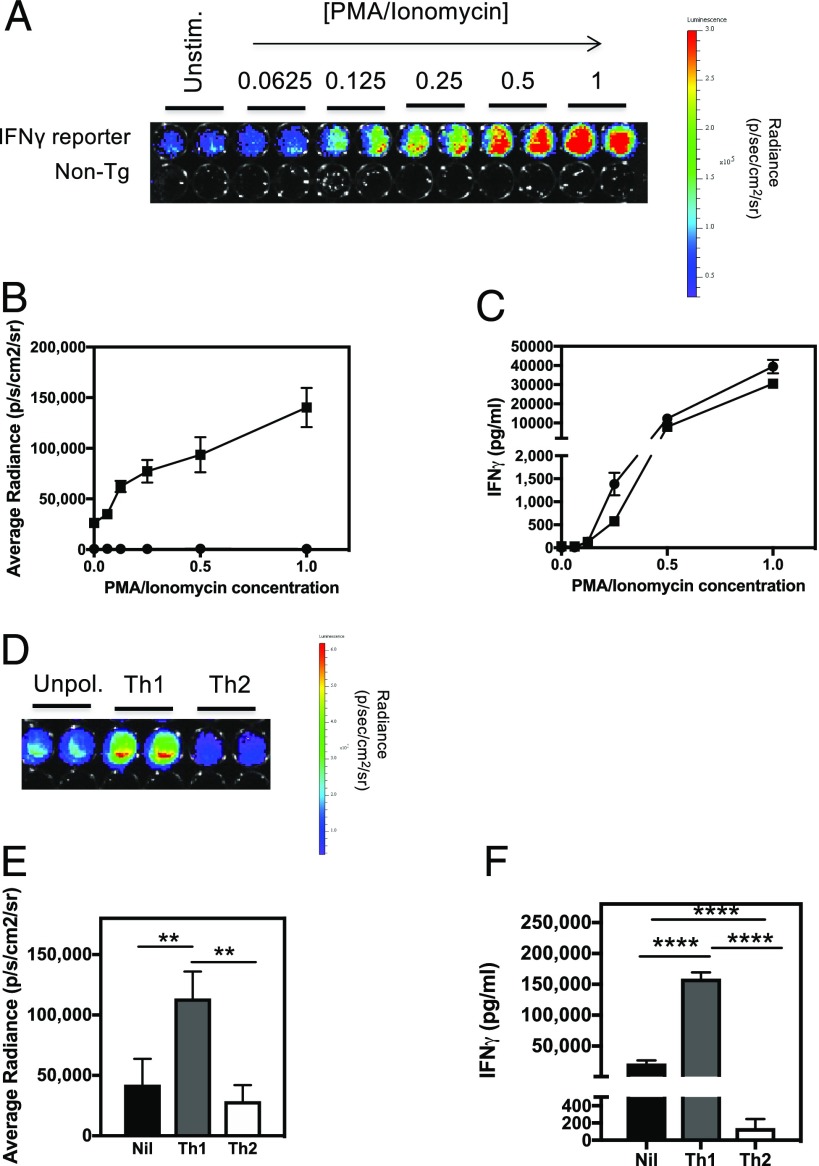
Bioluminescence signal in IFN-γ reporter transgenic is linked to IFN-γ production. (**A**) Splenocytes from IFN-γ reporter (*n* = 8) and nontransgenic (*n* = 8) mice were stimulated with increasing doses of PMA and ionomycin for 24 h. A concentration value of 1 represents 50 ng/ml of PMA and 1 μg/ml Ionomycin. d-Luciferin was added to wells of the culture plate at a concentration of 150 μg/ml and imaged for bioluminescence. A representative image of the PMA and ionomycin dose response for an IFN-γ reporter and non-transgenic mouse is shown. (**B**) Average radiance for each dose of PMA and ionomycin in both IFN-γ reporter (squares) and non-transgenic (circles) cell cultures were calculated. (**C**) Cell culture supernatants were also removed for quantification of IFN-γ protein by ELISA. (**D**) Splenocytes from IFN-γ reporter mice (*n* = 6) were stimulated with anti-CD3 and anti-CD28 Abs and cultured with or without Th1- or Th2-polarizing cytokines for 72 h before bioluminescence imaging. A representative image of unpolarized, Th1-polarized, and Th2-polarized splenocytes is shown. (**E**) The average radiance for each culture condition was measured, and (**F**) IFN-γ protein levels in the culture supernatants were determined by ELISA. Error bars represent mean ± SEM. This experiment was repeated on two separate occasions. Statistical significance was determined using an unpaired *t* test. **p < 0.005, ****p < 0.0001.

Data presented in Fig. 1 suggest that IFN-γ is continuously produced in the lymphoid tissue of normal, healthy mice. We next sought to establish that the bioluminescence signal being detected was indeed reporting IFN-γ production. Splenocytes from IFN-γ reporter and nontransgenic control mice were stimulated for 24 h with increasing doses of PMA/ionomycin to induce T cell activation and IFN-γ production. The bioluminescence signal from the activated splenocytes was measured by IVIS (Fig. 2A, 2B) and shows that there is a steady increase in bioluminescence signal with increasing concentrations of PMA/ionomycin. This mirrors the steady increase in IFN-γ protein secreted by activated splenocytes (Fig. 2C). In addition, actively polarizing splenic T cells toward a Th1 or a Th2 phenotype by stimulation in the presence of polarizing cytokines and Abs resulted in increased and decreased bioluminescence under Th1- and Th2-polarizing conditions, respectively (Fig. 2D, 2E). Again, this pattern of bioluminescence mirrored the expected differences in IFN-γ protein production by these different cell populations (Fig. 2F).

### IFN-γ reporter transgenics can be used to monitor IFN-γ responses to infection in vivo

**FIGURE 3.**
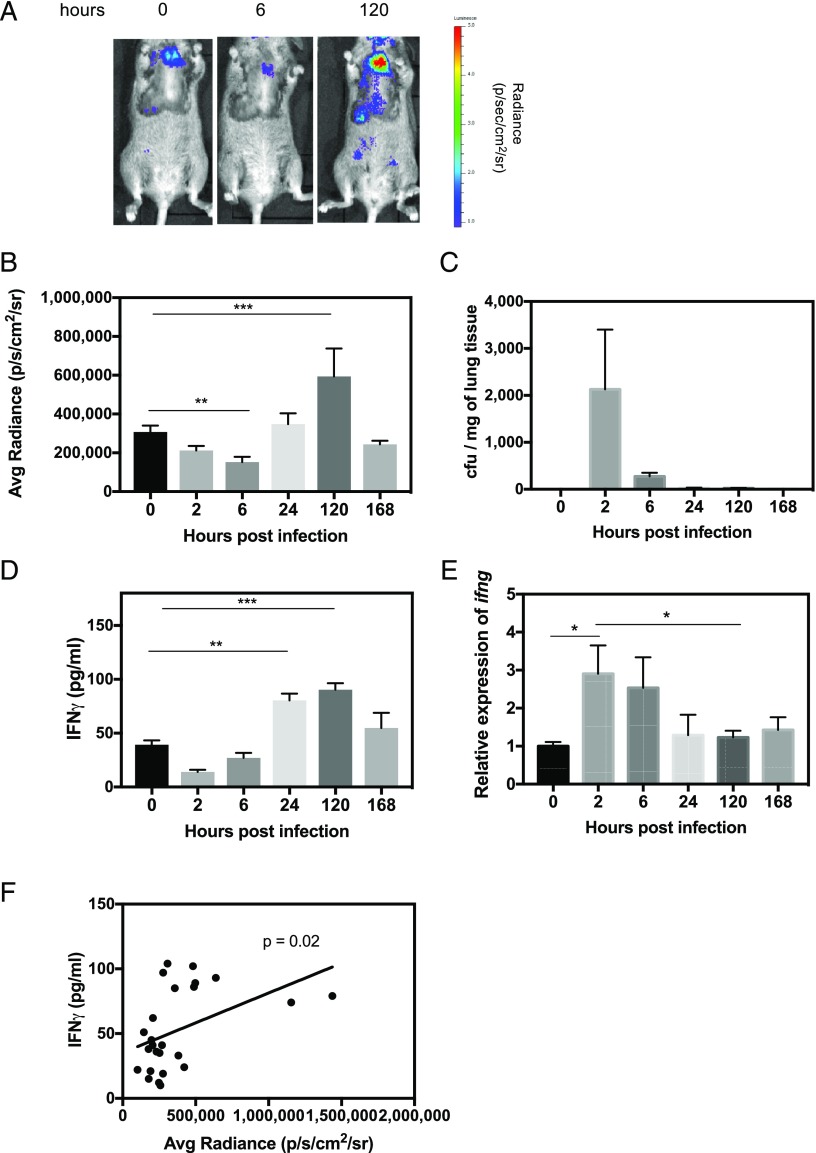
Reporter transgenic can be used to monitor IFN-γ responses in vivo in a model of *P. aeruginosa* lung infection. IFN-γ reporter mice were infected intranasally with 5 × 10^6^ CFU of *P. aeruginosa* (PA01). Mice were IVIS imaged at 0, 2, 6, 24, 120, and 168 h postinfection. (**A**) Representative images at 0, 6, and 120 h postinfection are shown. (**B**) At each time point, average radiance of the thorax of each mouse was measured, and a cohort of mice (*n* = 4) were culled for (**C**) determination of lung CFU burden, (**D**) lung IFN-γ protein quantification by ELISA, and (**E**) relative expression of *ifng* transcripts by real-time PCR. (**F**) Average radiance of the thorax was shown to correlate with amount of lung IFN-γ protein using the Pearson product-moment correlation coefficient. Error bars represent mean ± SEM. Statistical significance was determined using an unpaired *t* test. *p < 0.05, **p < 0.005, ***p < 0.0005.

Having established that bioluminescence in this model faithfully reports IFN-γ production and demonstrating that the bioluminescent signal from internal organs is capable of penetrating tissue for detection during in vivo imaging of a live animal, we next investigated whether this model could be used to follow the IFN-γ response to infection. We used a *P. aeruginosa* model of lung infection wherein mice were infected intranasally with a nonlethal dose and then monitored over the course of 7 d for changes in bioluminescent signal from the thorax (Fig. 3A, 3B). Bioluminescence decreased over the first 6 h of infection, peaked at day 5, and returned to baseline by day 7. This was in the context of mice having completely cleared the infection by this time point (Fig. 3C). To determine whether these changes in bioluminescence were a true reflection of the IFN-γ response in this model, cohorts of mice were culled at each time point, and lung tissue was harvested for IFN-γ measurement at the protein and transcriptional level. Bioluminescence correlated well with IFN-γ protein measurements, which also showed an initial decrease followed by a peak at day 5 (Fig. 3D, 3F). *Ifng* transcripts peaked at 2 h postinfection and decreased thereafter (Fig. 3E). These data further validate the reporter model. Correct targeting to the *ifng* gene in this reporter transgenic was achieved using a 93-bp 5′ homology arm and a 163-bp 3′ homology arm immediately upstream of *ifng* exon 1 and downstream of *ifng* exon 4, respectively. Thus we used extensive IFN-γ upstream and downstream regulatory sequence in a 174 kb BAC clone. As such, the expression faithfully mimics IFN-γ transcription. The fact that protein expression is to some degree more in step with bioluminescence than gene expression likely reflects specific nuances of the stability of the IFN-γ message.

### IFN-γ reporter transgenics for monitoring adaptive immunity

**FIGURE 4.**
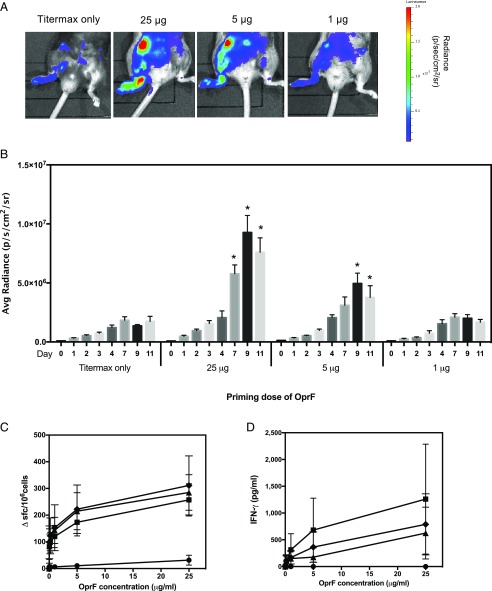
The adaptive T cell IFN-γ response to different priming doses of Ag can be detected by IVIS imaging in reporter transgenics. (**A**) IFN-γ reporter mice were immunized s.c. in the footpad with 25 μg (*n* = 9), 5 μg (*n* = 8), or 1 μg (*n* = 9) of the *P. aeruginosa* Ag OprF in TiterMax adjuvant or with TiterMax adjuvant alone (*n* = 7). A representative image for each group at day 9 postimmunization is shown. (**B**) Bioluminescence signal from the immunized footpad of each mouse was quantified on days 0, 1, 2, 3, 4, 7, 9, and 11. Data are from two independent experiments combined. (**C**) At day 11 postimmunization, DLN cells were harvested from mice that had been immunized with TiterMax only (circles) or 25 μg (squares), 5 μg (triangles), or 1 μg (diamonds) of OprF protein and assayed for IFN-γ production in response to different doses of OprF by ELISPOT and by (**D**) ELISA, using supernatants from DLN cells cultured with OprF protein for 3 d. Error bars represent mean ± SEM. For bioluminescence data, statistical significance between mice primed with Ag and those primed with adjuvant only was determined using an unpaired *t* test. *p < 0.05.

IFN-γ production ex vivo is often used as a readout for recall responses to Ag in models of infection and immunization. However, it is difficult to measure IFN-γ responses occurring in vivo at the point of the initial primary immune response to Ag. We used IFN-γ reporter mice to follow the primary IFN-γ immune response to different doses of the *P. aeruginosa* Ag, OprF, given with adjuvant. Mice were immunized in the footpad, and the bioluminescence signal was monitored over the course of 11 d of the primary immune response (Fig. 4A, 4B); the T cell response in this context is initially localized to the popliteal lymph node. Mice that received adjuvant alone mounted a small IFN-γ response that increased steadily to day 7 before plateauing. The bioluminescence signal in these mice was indistinguishable from that in mice that had been primed with 1 μg OprF. However, it was possible to distinguish between mice that had received 25 or 5 μg of Ag. By day 7, the IFN-γ response in the footpad of mice that had received 25 μg OprF was significantly higher than in the other groups, and by day 9, those mice primed with 5 μg of protein also showed a higher bioluminescence signal than those primed with the lowest dose or adjuvant alone. These data contrasted with that seen for recall response to Ag, measured using draining lymph nodes (DLN) at day 11 postimmunization by IFN-γ ELISPOT and ELISA (Fig. 4C, 4D). By IFN-γ ELISPOT, all three priming doses of Ag gave a significantly higher IFN-γ signal than adjuvant alone and were equivalent to each other in the magnitude of their IFN-γ response. Bioluminescence picks up the adaptive recall immune response to Ag with similar sensitivity and reproducibility to the ELISPOT at priming doses of 5 and 25 μg of Ag (Fig. 4B, 4C), notwithstanding the caveat that bioluminescence can be assessed daily through the study (the advantage of this approach), with differences being identified at earlier time points, whereas the ELISPOT is conducted as a terminal one-off analysis at day 11.

### In vivo visualization of T cell activation and tissue infiltration using IFN-γ reporter transgenics

**FIGURE 5.**
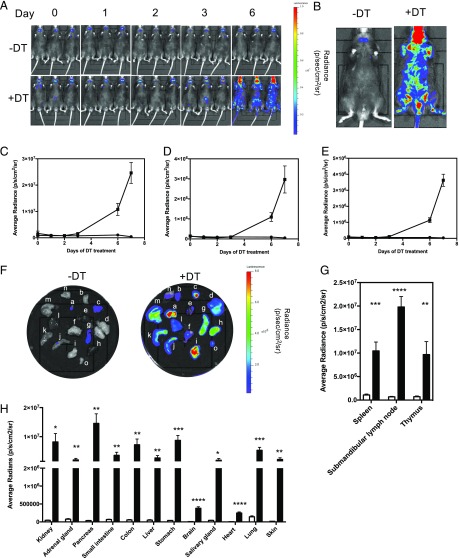
T cell activation and tissue infiltration in the Foxp3-DTR model can be tracked and visualized using the IFN-γ reporter transgenics. IFN-γ reporter mice that had been crossed onto the Foxp3-DTR transgenic line (*n* = 6) were given DT (1.25 μg/kg) on alternate days via i.p. injection. Another cohort of mice did not receive any DT (*n* = 6). Mice were IVIS imaged. (**A**) Representative images of untreated and DT-treated mice on days 0, 1, 2, 3, and 6 are shown. (**B**) Representative images of untreated and DT-treated mice at the end of the experiment (day 7) are shown. Average radiance of the neck (**C**), thorax (**D**), and abdominal (**E**) regions of each untreated (circles) and DT-treated (squares) mouse were measured. (**F**) All mice were culled at day 7, and submandibular lymph nodes (**a**), salivary gland (**b**), thymus (**c**), lung (**d**), heart (**e**), skin (**f**), spleen (**g**), kidney (**h**), pancreas (**i**), small intestine (**j**), colon (**k**), liver (**l**), stomach (**m**), brain (**n**), and adrenal glands (**o**) were dissected and IVIS imaged. Average radiance was calculated for (**G**) lymphoid tissue and (**H**) all other organs in untreated (white bars) and DT-treated (black bars) mice. Data shown are mean ± SEM. This experiment was repeated on two separate occasions. Statistical significance between DT-treated and untreated mice was determined using an unpaired *t* test. *p < 0.05, **p < 0.005, ***p < 0.0005, ****p < 0.0001.

The Foxp3-DTR model of conditional Treg depletion (19) is a well-characterized one in which loss of Tregs allows the activation of autoimmune T cells, leading to infiltration of many different organs and causing premature death. We crossed these mice onto the IFN-γ reporter line to determine whether the luciferase reporter could be used to follow the time course for the onset and location of disease in these animals. Tregs were depleted in one cohort of mice by the administration of DT. The increase in bioluminescence over time in these mice was followed by IVIS imaging (Fig. 5A) until the experimental endpoint at day 7 (Fig. 5B). The anatomical regions of the neck, thorax, and abdomen showed bioluminescence that increased significantly, and radiance from these areas was quantified over time (Fig. 5C–E). The data shows that the IFN-γ signal increases following Treg depletion. At the end of the experiment, internal organs were dissected and imaged ex vivo by IVIS (Fig. 5F). Quantification of the bioluminescent signal from each organ showed that increased IFN-γ production was greatest in lymphoid organs, the digestive tract, and the pancreas but that there was a significant increase in almost every organ analyzed (Fig. 5F–H). This suggests that it would be possible to use this reporter mouse to study disease development in almost any model of autoimmune disease.

## Discussion

During recent decades of research into T cell immunology in mouse models, a range of approaches have been developed to quantify Ag-specific immunity. The field has witnessed the progression through preference for measuring delayed hypersensitivity by tissue thickening and then [^3^H]thymidine incorporation assays, ELISPOTs, intracellular cytokine staining, and CFSE dilution. Our aim in this study was to add to this arsenal an in vivo, noninvasive assay that would offer a sensitive readout of adaptive, Th1 immunity in real time in live mice. The application of a bioluminescent reporter for cytokine promoter activation affords the potential for in vivo imaging across a time course using a simple IVIS chamber. This offers clear advantages in terms of ease of immune assays and also has important implications for animal welfare with reduced mouse numbers being used because the same immunized cohort can be followed and imaged longitudinally. Bioluminescent imaging of IFN-γ offers an approach to questions of comparative screening of T cell immune responses, whether comparing between vaccines, Ags, adjuvants, or inhibitory therapeutics. As an easily understandable shorthand, we have termed this reporter strain “Gammaglow.”

In the current study, we generated a construct based on a BAC carrying the murine *ifng* locus, but the approach can readily be adapted to any other cytokine. An obvious approach might be to generate the resource of a series of cytokine reporters encompassing IL-4, IL-17, and IL-10 and carrying bioluminescent reporters emitting at nonoverlapping wavelengths. Although the BAC construct generated for this work yielded a strong luciferase signal, the corresponding enhanced GFP signal was poor, making it impossible, for example, to phenotype and sort responding cells by flow cytometry. It will be necessary in the future to look at exchanging the enhanced GFP cassette for an alternative fluorescent protein. Furthermore, although the signal from lymphoid organs was sufficient for IVIS imaging in vivo for our studies, the use of these mice would be further enhanced by crossing onto an albino background.

During initial characterization of the reporter line, it was noted that at baseline, housed in a specific pathogen-free facility, transgenics showed a significant IFN-γ signal from spleen, submandibular lymph nodes, thymus, and lung (Fig. 1). Further studies will be needed to determine the cellular origins and driver of this IFN-γ responsiveness; this may constitute an ongoing, homeostatic response to environmental or microbiota antigenic stimulation. An interesting finding from the *P. aeruginosa* infection studies was that there was an initial drop in this baseline IFN-γ signal. This observed reduction in background IFN-γ signal may be because of reduced drive from the lung microbiota because of competition for the lung niche.

In initial studies to validate the bona fides of the Gammaglow reporter mice for reporting IFN-γ responsiveness, we compared PMA/ionomycin in vitro stimulation of splenocytes in terms of bioluminescence relative to actual IFN-γ ELISA. Detection of an IFN-γ response was more sensitive by bioluminescence than by ELISA.

An important application for cytokine bioluminescence strains such as Gammaglow is in monitoring responses to infection, including the efficacy of vaccine strategies. In the current study, we demonstrate this principle through the example of lung infection by *P. aeruginosa*. Vaccine studies indicate that a T cell IFN-γ response is a component of protective immunity to *P. aeruginosa* (21). Noninvasive IVIS imaging detected the IFN-γ response to *P. aeruginosa* from ∼24 h postinfection. The novelty of these challenge studies is thus not in the ability to study *P. aeruginosa* infection per se but the capacity to identify activation of an in vivo IFN-γ response as early as 24 h postinfection, offering the facility to monitor the response in real time from initial recognition and cellular activation. This has important implications for assessing correlates of protection to a pathogen of considerable current concern with respect to antimicrobial resistance and the need for vaccine programs. Because of the relative ease with which extremely bright, bioluminescently labeled pathogens can be engineered, there is clear potential in this study to combine these approaches to allow simultaneous IVIS imaging using different luciferases for measurements of pathogen load/spread and the host adaptive response to it. Using Gammaglow mice to monitor the response in vivo to footpad immunization with *P. aeruginosa* Ag in adjuvant, we found that adjuvant alone elicited an IFN-γ signal, but the Ag-specific response could be detected as early as 7 d after immunization. The TiterMax Gold adjuvant used would indeed be expected to enhance baseline IFN-γ because it contains CRL-8300 copolymer in squalene.

A key driver for our efforts to generate bioluminescence cytokine reporter mice has been the need for tools for the characterization of autoimmune pathogenesis and its control. In an age of clinical trials using anti-cytokine biologics, there has been considerable need to appraise the differential contributions of effector cytokines to various autoimmune pathologies in preclinical studies using murine models. Fontolizumab, a therapeutic monoclonal targeting IFN-γ, has been trialed in a number of clinical autoimmunity settings, including phase II trials (22). Even for diseases such as multiple sclerosis and type 1 diabetes in which attention has focused on a pathogenic role of IL-17, there is much evidence for involvement of IFN-γ^+^ cells (23, 24). As a means of investigating the development of autoimmune disease in vivo, we made use of the fact that Foxp3-DTR mice develop spontaneous, systemic, lethal autoimmunity on depletion of Tregs by DT treatment (19). When Kim and colleagues (19) injected mice with DT from birth and then on alternate days, mice became moribund by day 27 with H&E staining showing intense mononuclear infiltrates in liver, lung; and skin. By flow cytometry, a majority of deregulated T cells had become activated as measured, for example, by Ki-67 expression. Analysis of autoimmune etiology would be greatly aided by the ability to track (and then characterize) the appearance of activated, autoimmune cells at disease sites early in the process. To investigate the feasibility of this approach, in this study, we treated Foxp3-DTR mice with DT. By in vivo IVIS imaging, deregulation of an autoimmune IFN-γ response was apparent as early as day 6. Thus, in addition to the prior understanding that Treg-deregulated mice show florid mononuclear infiltrates (by H&E histology) in diverse organs, we were now able to show that, starting from a strong signal in spleen, thymus, and submandibular lymph nodes and then other sites including pancreas, ileum, colon, heart, lung, and brain, this approach can be used to track the appearance of autoimmune IFN-γ^+^-activated cells, which can then be used in studies of autoimmune mechanism. Analyzing the autoimmune response at a single, fixed endpoint of 4 wk by post mortem H&E staining of infiltrated tissues has been the standard in Treg depletion studies. This new model offers the significant advance of being able to follow the kinetics of the earliest autoimmune events, shown in this study as detectable from day 6, and also allowing precise quantification of the autoimmune infiltration into each organ in the face of Treg deregulation. These data have not been readily attainable by other approaches. Using data from IVIS tracking of autoimmunity in this new model, researchers will be able to track and characterize the earliest events in the autoimmunity of deregulated mice in affected organs.

In summary, using the example of Gammaglow IFN-γ–BAC luciferase reporter transgenic, we demonstrate in this study the principle of using bioluminescence cytokine reporters as a means of tracking IFN-γ immunity in Ag discovery as well as autoimmune or infectious disease settings. This offers the potential of noninvasive, longitudinal analysis of immune responses over time as well as identification of anatomical sites of response. Importantly, it also offers ethical advantages through substantial reduction in numbers. The former Editor-in-Chief of *The Journal of Immunology*, Pamela Fink, added a new section to the Table of Contents of *The Journal of Immunology*, called *Novel Immunological Methods* (25). She emphasized the need for peer review journals to work with researchers seeking to describe, “novel reagents, new genetically manipulated lines of mice, and innovative techniques in sufficient detail and with the appropriate experimental validation” (25). She proposed that this section would provide a home for such work and provide “a good resource for those who want to apply such new technology to solve pressing immunological issues” (25). This new approach to type 1 immunity analysis offers just such a resource.
